# A multi-label approach to target prediction taking ligand promiscuity into account

**DOI:** 10.1186/s13321-015-0071-9

**Published:** 2015-05-30

**Authors:** Avid M Afzal, Hamse Y Mussa, Richard E Turner, Andreas Bender, Robert C Glen

**Affiliations:** Department of Chemistry, Centre for Molecular Informatics, University of Cambridge, Lensfield Road, Cambridge, CB2 1EW UK; Department of Engineering, Computational and Biological Learning Lab, University of Cambridge, Trumpington Street, Cambridge, CB2 1PZ UK

**Keywords:** Multi-label classifications, Ligand promiscuity, Probabilistic classifier

## Abstract

**Background:**

According to Cobanoglu et al., it is now widely acknowledged that the single target paradigm (one protein/target, one disease, one drug) that has been the dominant premise in drug development in the recent past is untenable. More often than not, a drug-like compound (ligand) can be promiscuous – it can interact with more than one target protein.

In recent years, in *in silico* target prediction methods the promiscuity issue has generally been approached computationally in three main ways: ligand-based methods; target-protein-based methods; and integrative schemes. In this study we confine attention to ligand-based target prediction machine learning approaches, commonly referred to as *target-fishing*.

The *target-fishing* approaches that are currently ubiquitous in cheminformatics literature can be essentially viewed as single-label multi-classification schemes; these approaches inherently bank on the single target paradigm assumption that a ligand can zero in on one single target. In order to address the ligand promiscuity issue, one might be able to cast *target-fishing* as a multi-label multi-class classification problem. For illustrative and comparison purposes, single-label and multi-label Naïve Bayes classification models (denoted here by SMM and MMM, respectively) for *target-fishing* were implemented. The models were constructed and tested on 65,587 compounds/ligands and 308 targets retrieved from the ChEMBL17 database.

**Results:**

On classifying 3,332 test multi-label (promiscuous) compounds, SMM and MMM performed differently. At the 0.05 significance level, a Wilcoxon signed rank test performed on the paired target predictions yielded by SMM and MMM for the test ligands gave a p-value < 5.1 × 10^−94^ and test statistics value of 6.8 × 10^5^, in favour of MMM. The two models performed differently when tested on four datasets comprising single-label (non-promiscuous) compounds; McNemar’s test yielded *χ*^2^ values of 15.657, 16.500 and 16.405 (with corresponding p-values of 7.594 × 10^−05^, 4.865 × 10^−05^ and 5.115 × 10^−05^), respectively, for three test sets, in favour of MMM. The models performed similarly on the fourth set.

**Conclusions:**

The target prediction results obtained in this study indicate that multi-label multi-class approaches are more apt than the ubiquitous single-label multi-class schemes when it comes to the application of ligand-based classifiers to *target-fishing*.

**Electronic supplementary material:**

The online version of this article (doi:10.1186/s13321-015-0071-9) contains supplementary material, which is available to authorized users.

## Background

It is now widely acknowledged that the single target paradigm (i.e. one protein/target, one disease, one drug) that has been the dominant premise in drug development in recent past is untenable as both drug-like compound (ligand) and target protein can be promiscuous [1, 2]. More often than not, a ligand can simultaneously interact with multiple proteins in a human cell; this observation can also be true with target proteins [2, 3]. For example, according to Mestres [4], there is on average 6–7 annotated targets per drug in DrugBank [5]. It is, therefore, important that ligand (and protein) promiscuity is taken into consideration when developing *in silico* target protein prediction models. In this regard, significant efforts have been made in recent years in taking into account the promiscuity issue when devising *in silico* target protein prediction models [1–3, 6–9] (and references there in). The state-of-the-art methods that consider ligand (and protein) promiscuity when predicting target proteins can be broadly divided into three categories namely ligand-based [1, 3, 6, 7, 10, 11], target-structure-based [1, 3, 6, 8], and ligand-target-pair-based [1, 3, 6, 9]. In this study we confine attention to ligand-based machine learning approaches, commonly referred to as *target-fishing*.

The central idea that constitutes the nub of the ligand-based machine learning approach is that a new ligand sharing enough structural similarity to a set of reference ligands annotated against known target proteins has a high probability of showing activity against the predefined target proteins [6] (and references therein).

The *target-fishing* approach began to appear in the cheminformatics literature over the last decade and a half [10–21]. According to Rognan [6], the *target-fishing* methods all share three basic components: (a) a set of reference ligands represented in a descriptor/feature space are selected; (b) a screening procedure, such as a machine learning algorithm (for example, Bayesian classification scheme, which is the focus of the present work), is devised; and (c) the screening procedure determines whether a new compound is likely to share the same target protein as the reference ligands. In short, all this means: using a given activity dataset comprising a set of reference ligands, a set of target proteins and a bipartite activity relation between the targets and ligands in the two sets, a model is constructed such that for a new ligand the model returns the appropriate targets against which this ligand shows activity – we will come back to this and describe it in more concrete terms.

**Fig. 1:**
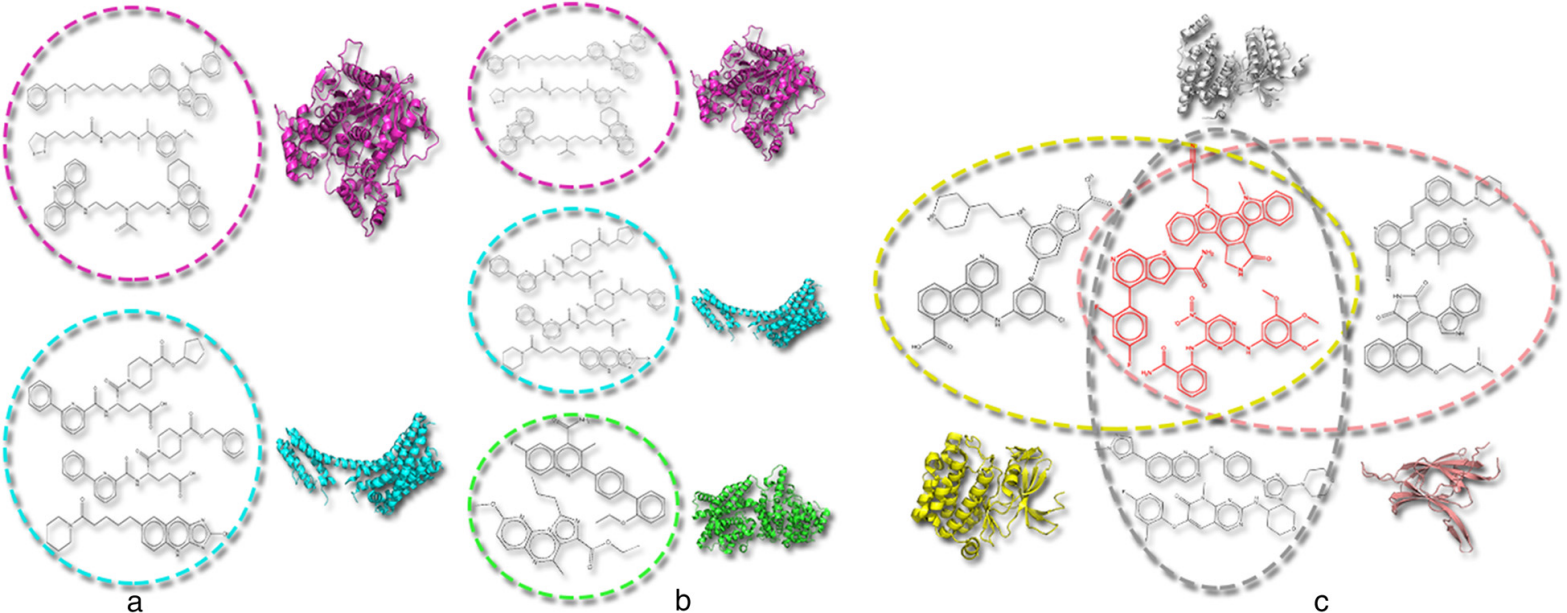
Different classification schemes. (**a**) Single-label binary classification scheme. Purinergic receptor P2Y12 (ChEMBL2001) shown in cyan and Butyrylcholinesterase (ChEMBL1914) shown in purple illustrate a binary classification problem. In binary classification, only 2 disjoint classes exist. Therefore in this classification scheme, |L| = 2 and |Y| = 1. (**b**) Single-label multi-class classification scheme. Phosphodiesterase 10A (ChEMBL4409) in green has been added to a single-label binary classification problem to form a single-label multi-class classification problem. That is to say, a single-label multi-class classification has more than two disjoint classes; hence, |L| > 2 (in this case |L| = 3) while |Y| = 1. (**c**) Multi-label multi-class classification scheme. Serine/threonine-protein kinase PIM1 (ChEMBL2147) in yellow, Protein kinase C delta (ChEMBL2996) in magenta and c-Jun N-terminal kinase 1 (ChEMBL2276) showed in grey, illustrate a multi-label multi-class classification. In multi-label classification problem, classes are not disjoint and compounds can belong to more than 1 class. Here, compounds shown in red belong to all 3 classes and have |Y| > 1 (in this case |Y| = 3). Furthermore, like the single-label multi-class classification problems, in this multi-label multi-classification scheme the value of |L| is bigger than 2 as we are dealing with more than two classes.

As far as we are aware, at the time of writing, the ligand-based machine learning approaches – with few exceptions (see the Previous work section) – utilised in cheminformatics explicitly or implicitly assume that the target proteins against which the reference ligands are annotated are mutually exclusive [3, 6, 10, 11, 15, 17, 22–24] (and references therein). It is assumed a ligand can (somehow) zero in on one single protein in the midst of the multitude of proteins in a human cell, which is the very questionable assumption noted above [1, 2, 4]. In machine learning (and also in statistics), this type of ligand-based target predicting approach can be viewed as a single-label multi-class classification problem, *vide infra*; this is schematically illustrated in Fig. 1. In contrast, as in this work, one might be able to take into account ligand promiscuity by casting the ligand-based target prediction task/approach as a multi-label multi-class classification problem. That is, the relevant target proteins for a certain ligand need not be mutually exclusive. Figure 1 shows an example of multi-label multi-class classification problem. The essence of “multi-label multi-class classification” and “single-label multi-class classification problem” will be covered and described in detail later in this section and also in the Materials and methods section.

In any event, in the light of the discussion in the preceding paragraphs the machine learning ligand-based target predicting approach (*target-fishing*) is basically a ligand-based classification problem [3, 6, 22–25], whereby a (machine learning) classifier is utilised to predict potential target protein(s) for a given ligand. Thus, developing an accurate – computationally efficient and conceptually appropriate – ligand-based classifier is an important research topic in cheminformatics. To this end, the nub of devising an efficient ligand-based classification model can amount to developing a mathematical algorithm that “learns” the chemical structure-biological activity relationships (if any) from a given set of reference ligand chemical structures, a predefined set of target proteins and a bipartite activity relation between the reference ligands and targets. Once the learning phase of the model/classifier building is completed, for a new compound the resultant classifier is expected to accurately predict relevant target proteins (in the preselected set of target proteins) against which this compound may show biological activity.

The ligand chemical structure is usually represented as a “vector” (descriptor/feature vector) whose elements, ideally, constitute the salient characteristics of the ligand for its interaction with potential target protein(s). There are a plethora of chemical structure representation schemes that have been suggested over the years [26, 27]. Simply one cannot predicate that a given representation of a chemical structure can capture all the subtleties intrinsic to a particular chemical structure of the ligand, which might be crucial for the biological effect that a ligand could induce on the relevant target proteins. Another source of uncertainty is the certitude that measurements of observable biological effects (and subsequently databases based on these observations) are inevitably noisy [28, 29]; this uncertainty can, in turn, introduce another layer of uncertainty in relating the chemical structure of the ligand with its observable activity against a target protein. It is, therefore, desirable to develop a ligand-based classification approach that takes into account these uncertainties. This deems a probabilistic classifier a good candidate for the task [19, 24, 30–37].

In more concrete terms, a ligand-based classifier can be viewed as an algorithm that appropriately associates ligand *j* denoted by a descriptor vector **x**_j_ with protein target(s) – often referred to as classes/labels, denoted by *l*_1_, …, *l*_*k*_, …, *l*_|*L*|_ – against which **x**_j_ may show activity. Henceforth, all the target labels are collectively denoted as set *L* = {*l*_1_, …, *l*_*k*_, …, *l*_|*L*|_}. Usually **x**_j_ is viewed as a “vector” defined on an *m*-dimensional descriptor space *χ*, where **x**_j_ = {*x*_*j*1_, …, *x*_*ji*_, …, *x*_*jm*_}; often the elements *x*_*ji*_ are assumed to represent the “relevant” chemical structure descriptors/properties of ligand *j* in relation to the targets. In the present work, *x*_*ji*_ are binary, representing the absence or presence of a chemical atom environment descriptors in the ligand.

A tacit assumption that is often made is that one has access to a representative dataset, *D*, that adequately captures the bipartite activity relation between the target proteins and reference ligand chemical structures: *D* = { (**x**_*j*_, *Y*_*j*_)}, *j* = 1, …, *N* denoting the *N* available data points, where **x**_*j*_ ∈ *χ* is as described before and *Y*_*j*_ ⊆ *L* refers to a set of targets against which **x**_*j*_ is known to be active.

In the literature [38–45], when |*Y*_*j*_| = 1, a classification model is referred to as a single-label classifier; but when |*Y*_*j*_| ≥ 2, the classification model is referred to as a multi-label classifier. Furthermore, a classification problem can also be called a binary classification problem if |*L*| = 2 (see Fig. 1a) and a multi-class classification problem when |*L*| > 2 (see Fig. 1b). Thus, a multi-class classification task can be categorised as a multi-label multi-class classification problem as depicted in Fig. 1c or as a single-label multi-class classification problem, (see Fig. 1b). For an extended and detailed account of the multi-label multi-class classification topic the reader may consult refs. [38–45].

Given *D*, the classification task amounts to “learning” or estimating a function (if one exists)

1 $$ f:\chi \to \Omega $$

which not only correctly associates **x**_*j*_ with its known label(s) *Y*_*j*_ , but also predicts the correct label(s) for a new ligand that is not included in the training dataset, *D*. (In the multi-label scenario, *Ω* is the power set of set *L*, whereas in the single-label approach, *Ω* = *L*.) In effect, our main task is to come up with a model that elucidates or captures the unknown underlying process that might have generated the observed phenomena, i.e. the dataset *D*, in the first place.

In Eq. 1 the function can denote a ligand-based deterministic or probabilistic classifier [30–37]. In the present work, attention has been confined to Naïve Bayes classifiers, which are probabilistic. In this case, both **x**_*j*_ and *Y*_*j*_ are random variables, but for notational simplicity in this work *l*_*k*_, *Y*_*j*_ and **x**_*j*_ denote both the random variables and the values they may assume. Furthermore, unless stated otherwise, the index *j* in **x**_*j*_, *Y*_*j*_ and *x*_*ji*_ and the index *k* in *l*_*k*_ are omitted for notational clarity.

As discussed in the Previous work section (see below), to our best knowledge – at the time of writing – the *target-fishing* approaches employed in cheminformatics (with a few exceptions) rely on the assumption that a given ligand can only interact with one target protein, i.e., |*Y*| = 1 [10–13, 15, 17, 22, 23]. In other words, these ligand-based target predicting methods, probabilistic or not, can be considered as single-label multi-class classification models [10, 11, 13, 15, 17, 22, 23]. In these methods a single-label multi-class classification model can be |*L*| induced binary (one–vs–all) classifiers [10, 12, 15], or just a single conventional multi-class classifier [11, 13, 17, 22, 23]. In this work we employed the latter classifiers (for our single-label multi-class classification problems), as they are more apt than the one–vs–all classifiers [23]. In any event, the high probability of a ligand interacting with more than one target protein – that is, |*Y*| ≥ 2 – in nature [1–4, 46–51] can render the single-label classification approach questionable as a *target-fishing* scheme.

In the light of our earlier discussion, when |*Y*| ≥ 2 (and, of course, |L| > 2), one may consider a ligand-based target prediction problem as a multi-label multi-class classification task. Since target proteins/classes are not necessarily mutually exclusive in the case of |*Y*| ≥ 2, a lone multi-label multi-class ligand-based model can, in principle, capture the underlying association (if any) between the chemical structure of a ligand and the set of labels *Y* ⊆ *L* denoting potential target proteins for the ligand. However, this principled approach is practically infeasible in our context. Nonetheless, there is nothing in principle to prevent one from approximating the ideal model by devising |*L*| induced binary classifiers that can associate a given ligand with its set of potential target proteins – providing that the available training set *D* is appropriately transformed (for a detailed account of training set transformation in the multi-label classification context, see ref. [39]). It is important to note that there are subtle, but crucial differences between the “conventional” induced binary classifiers (i.e., one-vs-all type classifiers) employed in single-label ligand-based models described earlier and the induced binary classifiers – that we have just described –henceforth referred to as “pseudo single-label” binary classifiers. This subtle issue is briefly commented on in the Methods section, but for a more detailed description, see ref. [38].

In our multi-label multi-class classification approach, |*L*| “pseudo single-label” binary classifiers were constructed, whereby the data transformation scheme utilised was *binary relevance* [39].

Classification approaches based upon Naïve Bayes markedly feature in the probabilistic classification models for *target-fishing* [10, 12, 13, 15, 19] (and references therein). For this reason, we concentrated on this particularly popular ligand-based classification model. The popularity of the Naïve-Bayes as a *target-fishing* tool can be probably attributed to the fact that building non-Naïve Bayes multi-class classifiers (be probabilistic or not) can become conceptually intricate or computationally demanding, or both [10, 11, 17–19, 22–24, 33–37]. The Naïve Bayes approach is: (1) probabilistic; (2) favourably scalable with *m*, |*L*|, and *N*, where *m*, |*L*| and *N* are as defined before; (3) computationally simple to implement; and (4) known to yield respectable classification results, despite the flimsiness of the rationale upon which the algorithm is based – that is, descriptors for a ligand are conditionally independent of each other given the class label. It is these characteristics that give the application of Naïve Bayes based *target-fishing* approaches an edge over other classification algorithms also employed for this purpose [19] (and references therein).

### Previous work

For more recent developments on *target-fishing* approaches, we refer the reader to refs. [1, 3] and [6]. To our knowledge, there were no research papers, at the time of writing, regarding the topic of comparing single-label and multi-label multi-class Naïve Bayes classifications for *target-fishing*. Michielan *et al*. [20] employed multi-label multi-class classification to classify cytochrome p450 substrates. The authors employed multi-label multi-class classification models based on SVM, M_L_K-Nearest-Neighbour, and Neural Network on a dataset of 580 cytochrome p450 substrates and seven isoforms. Hristozov *et al.* [21], also employing SVM, Neural Network, and M_L_K-Nearest-Neighbour methods [42], looked into classifying sesquiterpene lactones into seven tribes from the plant family Asteraceae. The two research groups compared the performance of single-label and multi-label models, and cautiously noted that multi-class classifiers based on the multi-label concept outperformed, or performed just as well as their corresponding single-label multi-class classifiers. However, their work did not feature the subject matter here: Naïve Bayes algorithms; besides, compared to ours their studies covered only seven targets. Wale and Karypis employed multi-label ligand-based classification methods [16]. Unlike our study, the the main thrust of Wale and Karypis’s work was about comparing how different multi-label ligand-based classifiers perform on classifying multi-label bioactivity datasets. Similarly Kawai *et al.*’s study [30] was confined to the analyses of the performance of a multi-label ligand-based SVM classifier; the single-label aspect did not feature in their work, nor did single-label and multi-label Naïve Bayes algorithms.

Closely following studies in text mining [52], we implemented and studied a ligand-based Naïve Bayes multi-label multi-class classification model (MMM) for *target-fishing*. We compared this classifier with a single-label multi-class ligand-based Naïve Bayes classification model (SMM) designed for the same purpose. Both classification models were built and tested on a bioactivity dataset extracted from the ChEMBL17 database [53], which was comprised of 308 protein target classes and 65,587 compounds.

In the following and preceding sections the words “ligand” and “compound” are used interchangeably. Also the terms “class”, “activity”, “label”, “target” and “target protein” are employed interchangeably. A single-label compound means that a compound is active against only one target, while a multi-label compound refers to a compound that is active against more than one target. A single-label dataset refers to a dataset containing only single-label compounds, whereas a multi-label dataset refers to a dataset comprising multi-label (i.e., promiscuous) compounds.

## Materials and methods

### Dataset

In order to construct and validate our MMM and SMM classification models, we used the ChEMBL17 database, which comprises more than 1 million annotated compounds and more than 10 million bioactivity records covering 9,000 targets. The dataset used for this study was a subset of ChEMBL17, which consisted of 65,587 unique compounds covering 308 human targets giving a total of 93,281 ligand-target pairs. Structures with reported activities (IC50/ki/kd/EC50) equal or better than 1 μM and confidence scores of 8 or 9 against human protein targets were selected. The confidence score represents the assay-to-target relationship in the ChEMBL database. It indicates the type of target assigned to a particular assay as well as the confidence that assigned target is the correct target for the assay. The range of the confidence score is from 0 to 9, where 0 represents uncurated data and 9 refers to a single protein target that has been assigned to the assay with high degree of confidence.

Although this bioactivity value represented highly potent compounds, given the increase in the size of ChEMBL17 database, it represented a sensible trade-off between biological activity and coverage of the chemical space. Only protein classes that contained between 120 and 720 data points were selected to ensure that the dataset was balanced.

Table 1 summarises our ChEMBL17 dataset *D*. Although, as it can be seen in the table, the majority of the compounds in the dataset were single-label compounds, there were a significant number of multi-label compounds (more than one-sixth of the total number of compounds) in our dataset. Hence, we believe, this was a suitable dataset for testing the hypothesis described in the Background section – that is, the multi-label multi-class approaches are more apt than the ubiquitous single-label multi-class schemes when it comes to the application of ligand-based classifiers to *target-fishing*.

**Table 1:** Distribution of the compounds and their associated protein targets in our ChEMBL17 dataset

Number of Annotated Targets	1	2	3	4	5	6	7	8	9	≥10
Number of Compounds	54,563	7,937	1,571	321	240	191	132	60	42	530
% of Total Number of Compounds	83.1 %	12.1 %	2.39 %	0.49 %	0.36 %	0.29 %	0.2 %	0.09 %	0.06 %	0.8 %

The table shows that 83.1 % of the total compounds were annotated against only one target protein, while the remaining 16.9 % of compounds were annotated against two or more protein targets; just over one-sixth of our ChEMBL17 dataset comprises multi-label ligands

**Fig. 2:**
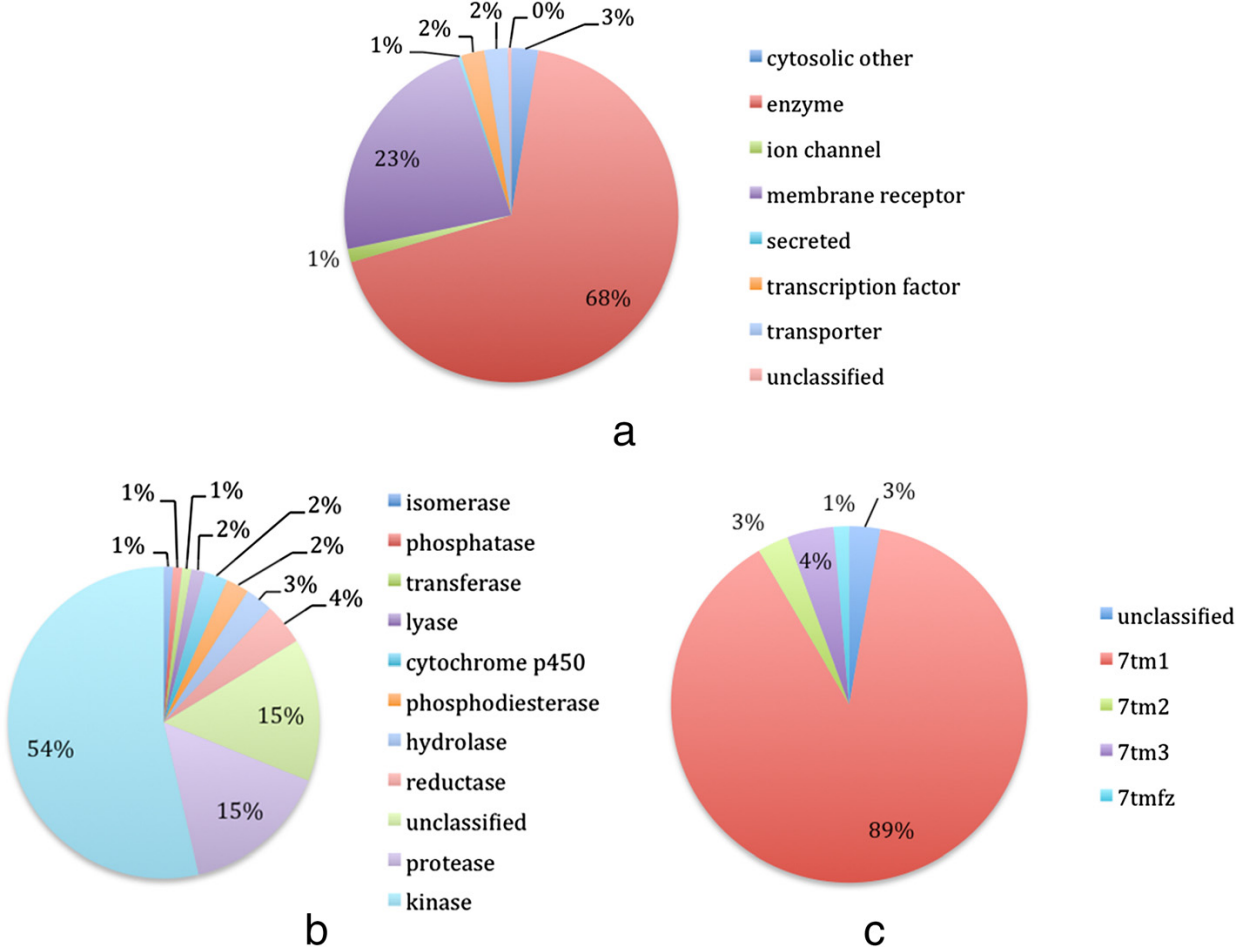
Protein target distribution in the ChEMBL17 dataset. (**a**) Protein target distribution among protein families in the ChEMBL17 dataset. (**b**) The distribution of protein targets in enzyme families. (**c**) The distribution of protein targets in membrane receptor families.

Table 2 and Fig. 2a depict the distribution of target proteins in different protein families. The majority of target proteins are categorised as enzymes and membrane receptors, with enzymes representing 67.8 % of all the protein targets/classes in our ChEMBL17 dataset, and membrane receptors constituting 23 % of it. Figure 2b depicts the distributions of the enzyme classes. A significant proportion of the enzyme families in our dataset consisted of the Kinase and Protease classes, with 54 % and 15 %, respectively. 7TM1 receptors constitute the bulk (89 %) of all the membrane receptor classes in our dataset (see Fig. 2c).

**Table 2:** Distribution of target proteins in different protein families in our ChEMBL17 dataset

	Enzyme	membrane receptor	transcription factor	ion channel	Transporter	cytosolic other	Unclassified	secreted
Number of Classes	209	71	7	4	7	8	1	1
% of Total Classes	67.85 %	23.05 %	2.27 %	1.29 %	2.27 %	2.59 %	0.32 %	0.32 %

90.8 % of the protein targets are enzymes and membrane receptors, with enzymes representing 67.8 % of all the protein targets, and membrane receptors constituting 23 %

A table showing the full list of ChEMBL IDs of targets and compounds per class is given as Additional file 1.

### Train and test sets

Our ChEMBL17 dataset was randomly split into two portions: 70 % of it as a training set and the remaining 30 % as a test set.

### Multi-label and single-label training set

The training set consisted of 45,911 compounds and their labels, whereby some of the compounds had more than one label. This set, which contained both single-label and multi-label compounds, was utilised to construct the multi-label multi-class model (MMM). The single-label multi-class model (SMM) was built on a single-label dataset, which was generated from the 45,911 compounds and their labels by simply associating each compound with only one label. If the compound was reported to be active against more than one target, the highest measured bioactivity (for the compound) was retained.

### Multi-label and single-label test set

The remaining 30 % of our ChEMBL17 dataset contained 19,676 test compounds. Among these, 16,344 compounds were single-label while 3,332 compounds were multi-label. This gave us two sets of test datasets: A single-label test set comprising 16,344 single-label compounds, and a multi-label test set consisting of 3,332 multi-label compounds.

The asymmetric abundance of the Kinase, Protease and 7TM1 classes in the dataset (see Table 2 and Fig. 2) allows us to generate three more datasets out of the original single-label test dataset (henceforth referred to as the “Global dataset”): one dataset comprising of Kinases; a dataset containing only Proteases and a 7TM1 dataset. This in turn allowed us to validate the two classification models (SMM and MMM) further, and more comprehensively scrutinise our hypothesis proposed in this work.

In passing, we only partitioned the original single-label test dataset into subsets because the number of single-label test compounds were not only 5 times (or more) larger than the number of multi-label test compounds contained in the multi-label test, but were also well distributed over the 308 target proteins constituting our predefined set of class labels.

### Compound descriptors

Compounds were standardized prior to fingerprint generation by ChemAxon’s Standardizer [54] using the options “Remove Fragments”, “Neutralize”, “Remove Explicit Hydrogen” and “Tautomerize”. Extended Connectivity Fingerprints (ECFP) were employed to describe compound structures [55–58]. ChemAxon’s Java API [54] was utilized to generate fixed-length ECFP_4 binary fingerprints with a length of 1,024 bits.

### Methods

In this section we briefly describe the single-label and multi-label multi-class Naïve Bayes algorithms that were employed in this study.

### Naïve Bayes

According to the Naïve Bayes assumption, the descriptors {*x*_1_ , . . . , *x*_*m*_} constituting the elements of the descriptor vector **x** representing the ligand are assumed independent given the class label *l* [19]. In this setting, a choice of *f* (in Eq. 1) can be the class posterior probability p(*l* | **x**), where p(*l* | **x**) can be expressed as [19]

2 $$ p\left(l\Big|\mathbf{x}\right)=\frac{{\displaystyle {\prod}_{i=1}^m}p\left({x}_i\ \Big|\ l\right)\ P(l)}{p\left(\mathbf{x}\right)} $$

where *P*(*l*) refers to the probability of the class label *l*. This term represents one’s state of knowledge about the class label before obtaining the data for the ligands. The term *p*(*x*_*i*_ | *l*) denotes the conditional probability for *x*_*i*_ given *l*, and *p*(**x**) is as defined below; *m* is as described before. In this study, *x*_*i*_ is binary – i.e., *x*_*i*_ ∈ {0, 1}. Comparatively, it is a simple affair to estimate *P*(*l*). Thus, in practice, estimating *p*(*l*|**x**) reduces to the estimation of ${\displaystyle {\prod}_{i=1}^m}p\left({x}_i\ \Big|\ l\right)$, i.e. the *p*(*x*_*i*_ | *l*)’s.

### Single-label Multi-class Naïve Bayes (SMM)

In the single-label multi-class Naïve Bayes model (SMM) employed in this work, where |*Y*| = 1 and |*L*| > 2, *p*(**x**) was expressed as $p\left(\mathbf{x}\right)={\displaystyle \sum_{l=1}^{\left|L\right|}}{\displaystyle \prod_{i=1}^m}p\left({x}_i\ \Big|\ l\right)\ P(l)$.

The class conditional probability *p*(*x*_*i*_ | *l*) was estimated as

3 $$ p\left({x}_i\ \Big|l\right) = \frac{\ 1+{n}_{il}^{+}}{2 + {n}_l} $$

where ${n}_{il}^{+}$ denotes the number of times that the *ith* descriptor *x*_*i*_ assumes the value 1 in class *l* and *n*_*l*_ is the number of instances in the training set belonging to class *l*. Here, *p*(*x*_*i*_ |*l*) was estimated using the Laplacian correction [19]. The prior distribution of each class *P*(*l*) was computed as

4 $$ P(l) = \frac{n_l}{N} $$

where $N$ denotes the total number of single-label training data points.

One classifier was built for each target protein *l* using Eqs. 2, 3, 4 and the compounds in the single-label training, which were annotated against this target protein only. For predicting potential target proteins for a new compound, SMM outputs |*L*| class/target posterior probability values – one for each class. The new compound is assigned to the class with the highest posterior probability value.

### Multi-label multi-class naïve Bayes (MMM)

The multi-label multi-class Naïve Bayes model (MMM), with |*Y*| ≥ 2 and |*L*| > 2, was implemented based on Wei et al. [52], where a *binary relevance* transformation [39] was utilised. However, any other appropriate transformation of the training set could have been employed [39]. Wei *et al*.’s approach is briefly described below for completeness. For a detailed account and more erudite description of what transforming the training set entails in the multi-label context, the reader is referred to ref. [39].

Using Eqs. 2, 3, 4 and a *binary relevance* transformation, |*L*| “pseudo single-label” binary classifiers, *H*_*l*_ : *χ* → {*l*, ¬ *l*}, were constructed – one for each unique label *l* in the set *L*. (The term “pseudo single-label” is as defined and described in the Background section and in the paragraph below.) In order to construct the |*L*| “pseudo single-label” binary classifiers, the original training dataset *D* was transformed into |*L*| datasets *D*_*l*_, where each *D*_*l*_ contains all the instances in *D*. Each compound in *D*_*l*_ is labelled active if it is labelled $l$ and otherwise labelled inactive by the class label ¬ *l*.

If all the compounds in the training dataset are single-label compounds, then a “pseudo single-label” binary classifier, *H*_*l*_ : *χ* → {*l*, ¬ *l*}, is nothing more than a one-vs-all binary classifier. However, in the ligand-based target predicting approach (*target-fishing*), a ligand needs not be a single-label compound. This means the binary classifier, *H*_*l*_ : *χ* → {*l*, ¬ *l*}, generated in our multi-label multi-class classification is not strictly a one-vs-all single-label classifier – hence, the attribute “pseudo single-label”. Obviously, one might more aptly call our *H*_*l*_ : *χ* → {*l*, ¬ *l*} a “pseudo one-vs-all” binary classifier. The reader is referred to ref. [38, 39, 52] for more details on this topic.

To predict the appropriate class labels (potential target proteins) for a new test compound **x**, the multi-label multi-class classification scheme – based on the “pseudo single-label” binary approach described earlier – outputs the union of the labels predicted by the |*L*| classifiers, *Z*:

5 $$ Z={\displaystyle \underset{l\ \in L}{\cup }}\left\{l:{H}_l\left(\mathbf{x}\right)\ge \kern0.5em {p}_{threshold}\right\} $$

where *H*_*l*_(**x**) denotes *p*(*l*|**x**) for compound **x**. Here *p*(*l*|**x**) was computed via Eq. 2, where *p*(**x**) and *p*(*l*) were given by $p\left(\mathbf{x}\right)={\displaystyle \prod_{i=1}^m}p\left({x}_i\ \Big|\ l\right)\ P(l) + {\displaystyle \prod_{i=1}^m}p\left({x}_i\ \Big|\neg l\right)\ P\left(\neg l\right)$ and $p(l) = \frac{n_l}{N}$ , whereas $p\left(\neg l\right) = 1-\frac{n_l}{N}$, and *p*(*x*_*i*_ | *l*) and *p*(*x*_*i*_ | ¬ *l*) were estimated by using Eq. 3 - *mutatis mutandis*.

Cross-validation was used to tune the parameter *p*_*threshold*_ (see Model construction and testing section).

### Model evaluation schemes

We employed two evaluation schemes namely a “Recall–Precision” metric, and a scheme based on ranking the class posterior probability estimates for the test compound labels. The “Recall–Precision” metric was employed to evaluate MMM and SMM performances on classifying single-label test compounds. The ranking evaluation metric was utilised to assess MMM and SMM performances on classifying multi-label test compounds.

### “Recall–Precision” metric: evaluating MMM and SMM performance on single-label data

In the multi-label multi-class classification scenario, a class prediction made by a multi-label multi-class model (MMM) can be fully correct, partially correct or fully wrong. Hence, the evaluation schemes for MMM are more complicated than those employed for evaluating the generalisation ability of a single-label multi-class model (SMM), whose prediction can only be fully correct or fully wrong.

For MMM, recall and precision evaluation measures based on ref. [44] are widely employed in the machine learning literature; we followed suit:

6 $$ precision = \frac{1}{\left|T\right|}\ {\displaystyle \sum_{t=1}^{\left|T\right|}}\frac{\left|{Y}_t{\displaystyle \cap }{Z}_t\right|}{\left|{Z}_t\right|} $$

7 $$ recall = \frac{1}{\left|T\right|}\ {\displaystyle \sum_{t=1}^{\left|T\right|}}\frac{\left|{Y}_t{\displaystyle \cap }{Z}_t\right|}{\left|{Y}_t\right|} $$

where *T* denotes the multi-label test set which has |*T*| examples (**x**_*t*_, *Y*_*t*_), *t* = 1, …, |*T*|; *Y*_*t*_ (⊆*L*) denoting the set of labels to which **x**_*t*_ belongs; and ${Z}_t={\displaystyle \underset{l\ \in L}{\cup }}\left\{l:{H}_l\left({\mathbf{x}}_t\right)\ge \kern0.5em {p}_{threshold}\right\}$ represents the set of labels to which **x**_*t*_ is predicted to belong.

However, in the case of SMM, recall and precision values are computed (per class) as

8 $$ precision = \frac{TP}{TP+FP} $$

9 $$ recall = \frac{TP}{TP+FN} $$

where “*TP*” denotes the number of compounds that the model assigns to their actual target, (say) target A; “*FN*” refers to the number of compounds annotated against target A, but assigned to other targets, whereas “*FP*” represents the number of compounds whose associated target was wrongly predicted to be target A.

Evaluating the generalization ability of SMM (using Eqs. 8 and 9) on classifying single-label compounds is straightforward. However, the same cannot be said about MMM because in this case the classification predictions can be partially correct, fully correct or fully wrong. Thus, to make the comparison of the classification performance of the two models (MMM and SMM) on the single-label dataset at hand as equitable as possible, only the predicted class label in the top position (i.e., with the largest class posterior probability value) of the predicted set of class labels *Z*_*t*_ for **x**_*t*_ is considered as the predicted class label when computing |*Y*_*t*_ ∩ *Z*_*t*_| in Eqs. 6 and 7. This means the so-called rejection option/ threshold value was not taken into consideration. (In both MMM and SMM, a class prediction resulting in a tie – two or more classes are equally predicted – is arbitrarily assigned to one class.)

It should be noted that, while the “Recall-Precision” metric described above puts the “recalls” in Eq. 7 and Eq. 9 on equal footings, it heavily penalises the precision value in the MMM case as the denominators in Eqs. 6 and 8 indicate.

In passing, a comparison of the two models (SMM and MMM) utilising single-label datasets may seem to be vacuous. However, we point out that this is not necessarily the case: As described in the *Multi-label multi-class naïve Bayes* section, our MMM is not a mere combination of |*L*| single-label binary classifiers, each being a one-vs-all binary classifier trained on a single-label dataset that ignores possible overlaps among the target proteins at the outset. Furthermore, a single-label test dataset does not necessarily imply that each compound in the set is conclusively single-label. Even if it were, MMM, which is more powerful/complex than SMM by design, could still have a better chance (than SMM) of correctly identifying the protein against which the test ligand is supposed to be active. However, MMM is also equally more likely to yield false positives than SMM will.

### The ranking metric: evaluating MMM and SMM performance on multi-label data

The ranking metric utilised in this work evaluates MMM and SMM performance (on classifying multi-label compounds) equitably. This evaluation scheme works as follows:

For a given a test ligand **x**_*t*_

- Using Eq. 5 with *p*_*threshold*_ being set to zero, MMM computes |*Z*_*t*_| class posterior probability estimates. (Note that |*Z*_*t*_| = |*L*| when *p*_*threshold*_ = 0.) These |*L*| posterior probability values computed are then ranked in descending order, such that the class/labels with the largest class posterior probability value computed is defined to be in rank position 1; the class/label with the second largest class posterior probability value is placed in rank position 2; and so on.
- Similarly, SMM computes |*L*| class posterior probability values that are also ranked in descending order, whereby the class/label with the largest class posterior probability value obtained is defined to be in rank position 1; the class/label with the second largest class posterior probability value is placed in rank position 2; and so on.
- If the test ligand **x**_***t***_ is known to be active against |*Y*_*t*_| targets (that is, the ligand is annotated against |*Y*_*t*_| labels), the rank positions of these labels in the |*L*| rank positions described in (a) (see above) are accordingly paired with their corresponding rank positions in the |*L*| rank positions yielded by SMM, described in (b). This results in |*Y*_*t*_| paired label/target rank positions for the test compound **x**_*t*_.
- (a), (b) and (c) are repeated for each test ligand. This yields *M* paired class label rank positions, whereby $M={\displaystyle {\sum}_{t=1}^{\left|T\right|}}\left|{Y}_t\right|$, with |*T*| denoting the number of test data points (see the preceding section).
- The Wilcoxon signed rank test is utilised to test whether the two *M* label rank positions – returned by MMM and SMM for the |*T*| test ligands – are statistically different.

### Model construction and testing

Our ChEMBL17 dataset *D* was randomly split into two portions – 70 % of it as a training set, and the remaining 30 % as a test set.

Using the 70 % ChEMBL17 dataset allotted to training, the multi-label multi-class classification and single-label multi-class classification models based on the Naïve Bayes concept were generated, see *Methods*.

The multi-label multi-class classification model (MMM) was built on the whole training set – i.e., containing both single-label and multi-label compounds. The single-label multi-class classification model (SMM) was built only on single-label training set, which was generated from the multi-label training set by simply associating each compound with only one of its targets, whereby the target with the highest measured bioactivity was retained.

To compare the classification performance of the MMM and SMM (on the remaining 30 % of our ChEMBL17 dataset) we utilised the two evaluation schemes described in the previous section: the “Recall–Precision” metric and the ranking scheme. In the SMM there was no parameter to estimate to compute Eqs. 8 and 9. However, in the MMM, the “optimal” value of the *p*_*threshold*_ value (see Eq. 5) had to be estimated to compute Eqs. 6 and 7. This was achieved via 5-fold cross validation on the single-label training set. For all the results given in the following section the MMM algorithm performed best when *p*_*threshold*_ was set to 0.999.

**Fig. 3:**
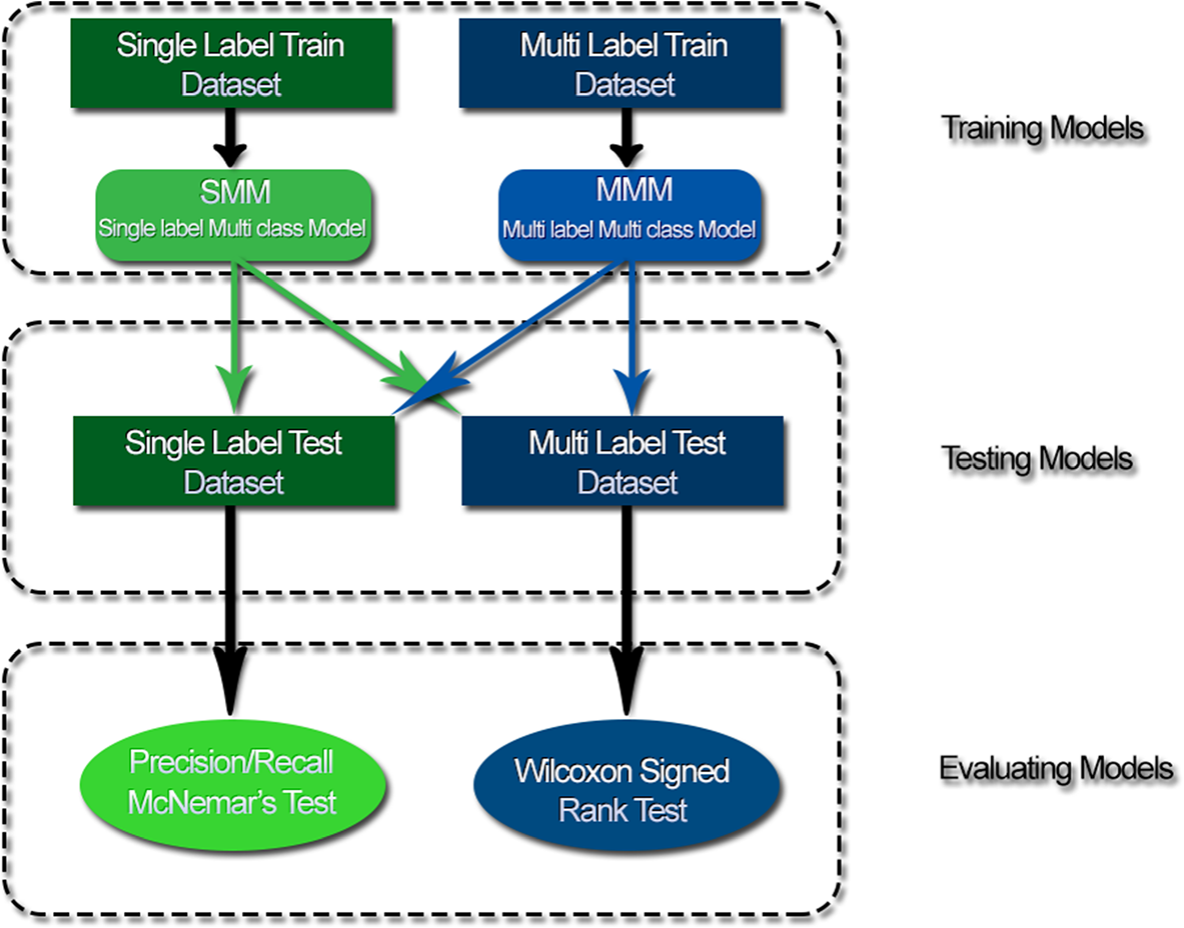
Workflow and datasets of the study. The workflow of this study and datasets used to test the hypothesis. Multi-label dataset, model (MMM) and evaluation procedures are shown in blue and single-label dataset, model (SMM) and evaluation steps are shown in green. The ChEMBL 17 dataset consists of single-label and multi-label compounds. This dataset was randomly split into 2 portions: 70 % as a training set and 30 % as a test set. The MMM was trained on the available training set whereas the SMM was trained only on single-label training set. This single-label training set was extracted from the multi-label training set by simply assuming that each compound belongs to only one target. Out of 19,676 test set compounds, 16,344 test compounds were single-label and 3,332 test compounds were multi-label. Hence, single-label test data set was built from 16,344 single-label test compounds while multi-label test set was built from 3,332 multi-label test compounds. SMM and MMM were tested on both single-label and multi-label test sets. To evaluate the performance of SMM and MMM models on single-label test set “Recall-Precision” and McNemar’s test were employed. On the multi-label test set, ranking scheme was utilised to compare the generalisation ability of the two models

Figure 3 summarises the workflow of this study and datasets used to test the hypothesis.

## Results and discussion

In the following analyses, in the first four subsections the test sets were comprised of single-label compounds, while in the fifth subsection the test set consisted of multi-label compounds. It is worthy of note that the class predictions (and the subsequent analyses) presented in this study were retrospective in the sense that the predicted targets were known beforehand.

### Classification performance on single-label test set

The two classification models, MMM and SMM, were tested on predicting the appropriate targets for the four single-label datasets: Global set; 7TM1 set; Kinases set; and Proteases set.

### Global test set

**Fig. 4:**
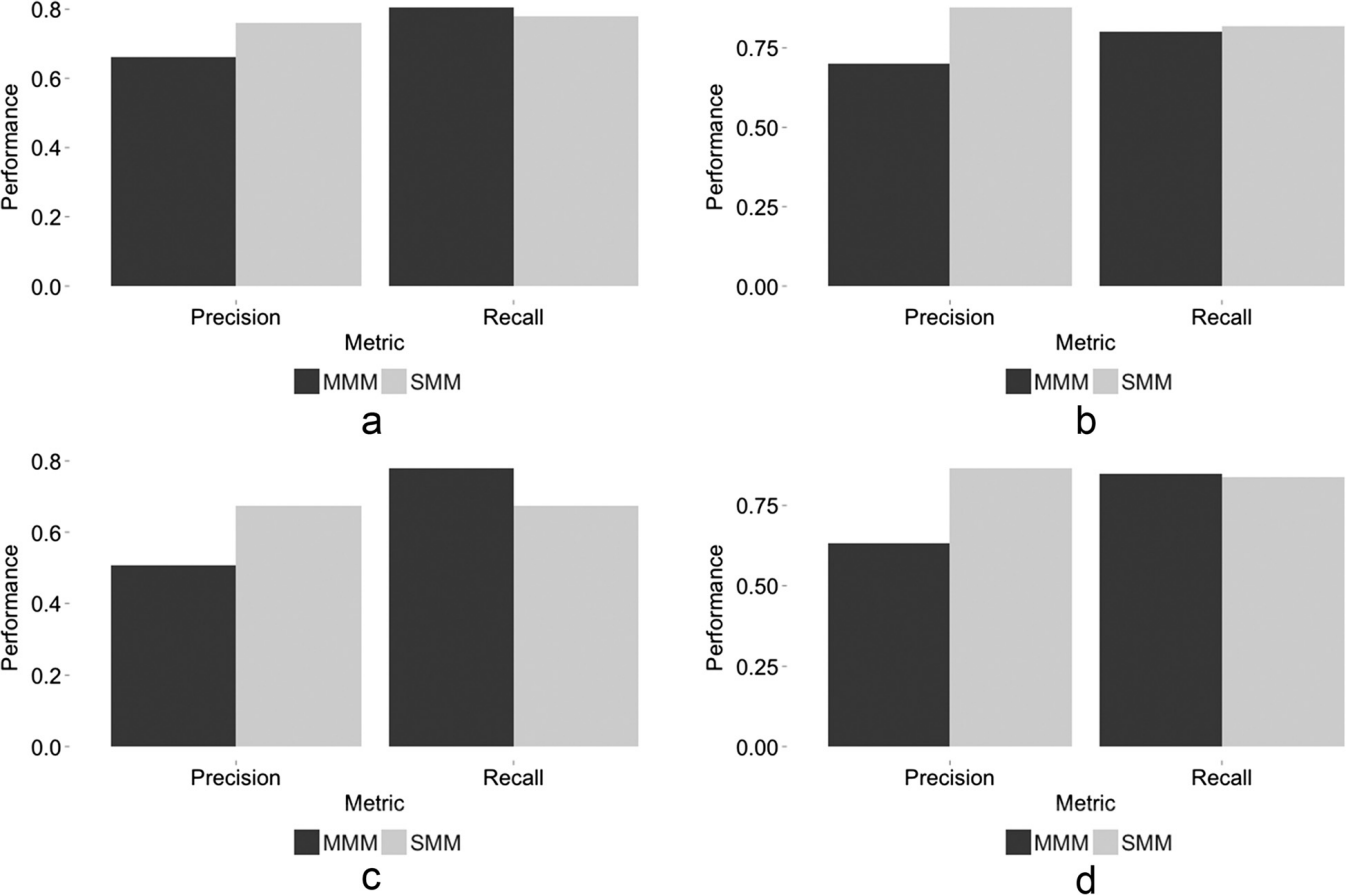
MMM and SMMM target prediction performance on test sets. (**a**) Bar plots of the recall and precision values shown in columns 2 and 3 in Table 3, the performances of the MMM and SMM models for 16,344 single-label ChEMBL17 test compounds covering 308 target proteins. (**b**) Bar plots depict the recall and precision values (Columns 2 and 3 in Table 4) that illustrate the performance of both models for 4,403 single-label ChEMBL17 test compounds covering 63 7TM1 target proteins. (**c**) Bar plots represent the recall and precision values (Columns 4 and 5 in Table 4) returned by the two classification models for 2,887 single-label ChEMBL17 test compounds covering 89 Kinase target proteins. (**d**) Bar plots denote the recall and precision values (Columns 6 and 7 in Table 4), the target prediction performance of the models for 1,927 single-label ChEMBL17 test compounds covering 31 Protease target proteins

MMM and SMM were tested on predicting the appropriate targets for 16,344 single-label test compounds distributed over 308 target proteins. Columns 2 and 3 in Table 3 demonstrate the target prediction performance of the two models for the single-label compounds in the global test set: SMM returned recall and precision values of 0.7805 and 0.7596 (Column 2), respectively; the corresponding recall and precision values yielded by MMM were 0.8058 and 0.6622 (Column 3), respectively. Figure 4a depicts the bar plots of the two sets of recall and precision figures in Columns 2 and 3 of Table 3.

**Table 3:** Recall and precision values returned by MMM and SMM on predicting the target proteins for single-label compounds in the global test set

Test Compounds	Single-label Compounds	
Models	SMM	MMM
Recall	0.7805	0.8058
Precision	0.7596	0.6622

**Table 4:** Recall and precision returned by MMM and SMM on predicting the target proteins for single-label test compounds annotated against single proteins in the 7TM1, Kinase and Protease protein families

Compounds	Single label compounds					
Protein Family	7TM1		Kinase		Protease	
Models	SMM	MMM	SMM	MMM	SMM	MMM
Recall	0.8176	0.8008	0.6726	0.7797	0.8376	0.8474
Precision	0.8783	0.7002	0.6741	0.5080	0.8666	0.6325

### 7TM1 test set

The test set consisted of 4,403 compounds distributed over 63 7TM1 proteins.

For this test set, SMM returned recall and precision values of 0.8176 and 0.8783 (Column 2: Table 4), respectively. The corresponding recall and precision values given by MMM were 0.8008 and 0.7002 (Column 3: Table 4), respectively. Figure 4b depicts the bar plots of these recall and precision pair values.

### Kinase test set

The dataset comprised 2,887 compounds annotated against 63 Kinases. For this test set, SMM gave recall and precision values of 0.6726 and 0.6741, (Column 4: Table 4), respectively; for the test set MMM yielded recall and precision values of 0.7797 and 0.5080 (Column 5: Table 4), respectively. The bar plots of the recall and precision pair values are shown in Fig. 4c.

### Protease test set

Here, the test set contained 1,927 compounds distributed over 31 Proteases.

For this test set, SMM yielded recall and precision values of 0.8376 and 0.8666 (Column 6: Table 4), respectively; and their corresponding recall and precision values obtained by MMM were 0.8474 and 0.6325 (Column 7: Table 4), respectively. Similarly Fig. 4d demonstrates the bar plots of these recall and precision pair values.

The recall figures reported for the Global, Proteases and Kinase test sets indicate that MMM returned better recall values than SMM. Only for the 7TM1 test set, the MMM classifier returned a recall value worse than the recall value yielded by the SMM classifier. However, SMM systematically gave better precision values compared to the precision values returned by MMM. These better precision values returned by SMM could be explained as an artefact due to the “Recall – Precision” evaluation metric employed: as described in the Method section, this evaluation scheme heavily penalises (see the denominators in Eqs. 6 and 8) the precision value returned by MMM.

Another possible explanation for the reason why SMM returned better (worse) recall (precision) values than those values obtained by MMM was that SMM and MMM were different in nature: SMM model was simpler than MMM as briefly described in the *Model evaluation schemes* section.

Further analysis of the single-label classification results revealed that MMM either statistically outperformed (or performed equally well as) SMM. For example, we statistically compared the number of single-label test compounds whose class labels were correctly predicted by MMM, but not SMM to the number of test compounds whose class labels were correctly predicted by SMM, but not MMM. McNemar's test [59] on these paired classification (and misclassification) results returned by the two models for the Global, 7TM1, and Proteases datasets yielded *χ*^**2**^ values of 15.657, 16.500 and 16.405 (with corresponding p-values of 7.5 x 10^−05^, 4.8 x 10^−05^ and 5.5 x 10^−05^), respectively – in favour of MMM. For the Kinases test set, the *χ*^**2**^ value was 2.485 (p-value of 0.1), which meant that MMM and the SMM performed similarly on this test set. Here and in the rest of the paper, the phrase “in favour of MMM” indicates that the number of test compounds whose labels were incorrectly predicted by the MMM, but not SMM < the number of test compounds whose labels were wrongly predicted by the SMM, but not MMM (see Additional file 2).

### Performance on multi-label test set

MMM and SMM algorithms were tested on predicting potential target proteins for 3,332 multi-label compounds, whereby each test compound **x**_*t*_ was known to be annotated against two or more target proteins (labels), but no more than 57 labels – i.e., |*Y*_*t*_| ∈ [2, 57]. This means each model was tested on predicting/identifying ${\displaystyle \sum_{t=1}^{\left|T\right|=3,332}}\left|{Y}_t\right|=5,656$ labels. The prediction performance of the two models was then compared by using the ranking metric scheme fully described in Section “*The ranking metric: evaluating MMM and SMM performance on multi-label data**”*. This resulted in 5,656 paired label rank positions.

**Fig. 5:**
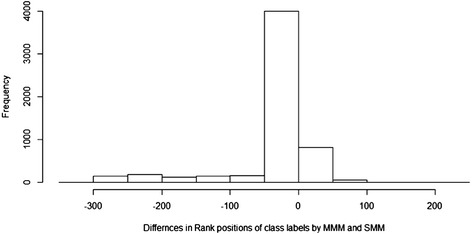
Histogram of pairwise differences of the labels predicted for test compounds annotated against two or more target proteins. The differences in performances of MMM and SMM algorithms on predicting the 5,656 labels is shown. On the *x-axis*, negative (positive) value indicates that the rank position, for a target, predicted by MMM (SMM) is higher than that by SMM (MMM). On the *x*-axis: zero denotes that MMM and SMM returned the same rank position for a target.

The rank positions predicted by SMM and MMM were similar for 3,336 labels. However, for the remaining 2,320 labels, models predicted different rank positions. For 886 labels (out of the 2,320 labels), SMM gave higher rank positions for the correct labels than MMM did, whereas MMM yielded better label rank positions for the other 1,434 labels. This is clearly depicted by the histogram in Fig. 5. Furthermore, at a 0.05 significance level, a Wilcoxon signed rank test performed on the 5,656 paired label rank positions gave a *p*-value < 5.1 × 10^−94^ (test statistics of 6.8 × 10^5^), which indicates that the two models performed differently with respect to the multi-label test dataset employed.

All these analyses suggest that MMM statistically generalises better than SMM based on the ChEMBL17 dataset utilised. Thus, one could argue that the *target-fishing* results yielded by our multi-label and single-label models certainly – albeit statistically – lend support to the argument against the single-target paradigm and *target-fishing* methods that are based on this paradigm.

## Conclusion

In this work two *in silico* ligand-based target prediction models – single-label multi-class and multi-label multi-class Naïve Bayes classifiers – were constructed and tested on a large dataset of bioactivity data extracted from the ChEMBL17 database. This dataset was randomly split into two portions: 70 % as training set and 30 % as test set. The training set was converted to single-label and multi-label training sets. The multi-label multi-class classification model (MMM) was built on multi-label training set while single-label multi-class classification model (SMM) on single-label training set. Furthermore, out of 19,676 test compounds, 3,332 compounds were multi-label (multi-label test set) while 16,344 compounds were single-label (single-label test set).

Statistically, MMM significantly outperformed its corresponding SMM on predicting the appropriate target proteins for 3,332 ChEMBL17 test compounds annotated against two or more (out of 308) target proteins. A Wilcoxon signed rank test performed on the classification results returned by SMM and MMM for the test set gave a *p*-value < 5.1 × 10^−94^ (test statistics of 6.8 × 10^5^), which indicated that the two models performed differently based on the dataset employed.

When tested on four (Global, 7TM1, Proteases and Kinases) datasets, each comprising ChEMBL17 only single-label test compounds, the MMM also statistically outperformed the SMM on three out of the four datasets. McNemar's test on paired MMM and SMM classification results for the global, 7TM1, and Proteases datasets yielded *χ*^**2**^ values of 15.657, 16.500, and 16.405, respectively (with corresponding p-values of 7.594 x 10^−05^, 4.865 x 10^−05^ and 5.115 x 10^−05^, respectively) – in favour of the MMM. The *χ*^**2**^ value was 2.485 (with a p-value of 0.115) for the fourth test set (the Kinases test set), which meant that MMM and SMM performed similarly. When the “Recall-Precision” evaluation metric was utilised, MMM returned better (worse) recall (precision) values compared to those values obtained by SMM (see Table 4).

The target prediction results obtained are in line with the hypothesis set out within this study, i.e., it is not appropriate, nor is it adequate to universally employ single-label multi-class ligand-based classification approaches for *target-fishing*. Thus, based on the datasets utilised in this work, one may conclude that out of the two classification approaches (SMM and MMM) tested, the multi-label multi-class model (MMM) is robust and more apt (and should be utilised) for ligand-based *target-fishing* purposes – the subject matter in this study.

## Additional files

Additional file 1:
**Full list of ChEMBL IDs of targets and compounds per class.**
Additional file 2:
**McNemar’s test result for single-label test sets.**

## Abbreviations

SMM: Single-label multi-class model
MMM: Multi-label multi-class model
SEA: Similarity ensemble approach
PASS: Prediction of activity spectra for substances
