# Supplementary material for: A multi-label approach to target prediction taking ligand promiscuity into account
Source: J Cheminform. 2015 May 30;7:24. doi: 10.1186/s13321-015-0071-9 (PMC4461803; doi:10.1186/s13321-015-0071-9)
Supplement: Additional file 2: — McNemar’s test result for single-label test sets. [file 13321_2015_71_MOESM2_ESM.docx]

**Supplementary materials.**

**Table 1 – McNemar’s test result for single-label test sets.**

|  | Global | | 7TM1 | | Kinase | | Protease | |
| --- | --- | --- | --- | --- | --- | --- | --- | --- |
|  | MMM  CorrectM | MMM  IncorrectM | MMM  CorrectM | MMM  IncorrectM | MMM  CorrectM | MMM  IncorrectM | MMM  CorrectM | MMM  IncorrectM |
| SMM  CorrectS | 12394 | 178 | 3406 | 16 | 1984 | 91 | 1467 | 21 |
| SMM  IncorrectS | 262 | 932 | 50 | 182 | 70 | 327 | 58 | 145 |
| McNemar's Test | 15.657 | | 16.500 | | 2.485 | | 16.405 | |
| p-value | 7.594 x 10^-05^ | | 4.865 x 10^-05^ | | 0.115 | | 5.115 x 10^-05^ | |

IncorrectS = the number of test compounds whose labels were incorrectly predicted by SMM;

IncorrectM = the number of test compounds whose labels were incorrectly predicted by MMM;

CorrectS = the number of test compounds whose labels were correctly classified by SMM;

CorrectM = the number of test compounds whose labels were correctly classified by MMM.

McNamara’s test was utilized to analyse the performance of both models. The test was performed (on the number of test compounds whose label were misclassified by MMM, but not SMM and the number of test compounds whose label were misclassified by SMM, but not MMM) for global, 7TM1, Kinases and Proteases datasets. As it can bee seen, at the 0.05 significant level, McNemar’s test yielded 15.6568, 16.5 and 16.4051 χ^2^ value for global, 7TM1 and Proteases test sets in favour of MMM. MMM and SMM models performed similarly on Kinase test set and the χ^2^ value was 2.4845. Note that “in favour of MMM” means that the number of test compounds whose labels were incorrectly predicted by MMM, but not SMM < the number of test compounds whose labels where wrongly predicted by SMM, but not MMM.
